# Primary Varicella or Herpes Zoster? An Educational Case Report From the Primary Care Clinic

**DOI:** 10.7759/cureus.23732

**Published:** 2022-04-01

**Authors:** Ana K Gómez-Gutiérrez, Areli A Flores-Camargo, Andrea Casillas Fikentscher, Eder Luna-Ceron

**Affiliations:** 1 Department of Clinical Sciences, Tecnológico de Monterrey, Monterrey, MEX

**Keywords:** vaccine-preventable disease, primary care medicine, herpes zoster, adult-onset, varicella vaccine, varicella zoster virus infection

## Abstract

Varicella-zoster virus is a pathogenic virus that can present itself as a primary infection or secondary infection, also known as herpes zoster. Recently, there has been a re-emergence of this vaccine-preventable disease due to gaps in vaccination. Primary varicella in immunocompetent adults is highly uncommon, and it could result in severe complications within this population. Given this delicate scenario, family physicians should be well trained to recognize the characteristic cutaneous lesions of varicella and dictate adequate management for these patients to obtain the best possible outcome and prevent life-threatening complications.

We present the case of a 43-year-old immunocompetent woman with the onset of a generalized pruritic dermatosis characterized primarily by the presence of macules, vesicles, and crusts. The patients' lesions were compatible with primary varicella, and serological studies confirmed the diagnosis. Given the absence of acute complications in this individual, supportive treatment and close follow-up were the therapeutic modalities. This article focuses on the educational discussion of the primary differential diagnosis, evaluation for possible complications, and management of this uncommon clinical scenario. We also reinforce the importance of immunization in preventing re-emergent diseases as a critical element within primary care management.

## Introduction

The introduction of vaccination has become a breakthrough in global health. In this regard, vaccines have provided coverage for several lethal diseases [1]. Unfortunately, the rate of vaccination has decreased within a couple of years [2]. For example, according to the World Health Organization (WHO), Mexico's vaccination rate has declined consistently since 2018 [3]. This phenomenon affects low-income countries and developed countries, such as seen in recent outbreaks in the United States [4,5]. In this sense, vaccine refusal has been associated with outbreaks of invasive *Haemophilus influenzae* type b diseases, varicella, pneumococcal disease, whooping cough [4], and measles [5]. The outcome has been an increase in the prevalence of cases of reportable and vaccine-preventable diseases all around the world. This significant increase is critical because it favors the risk of severe infections and poor clinical outcomes in populations where the incidence of these diseases was significantly reduced because of the vaccination era [6].

Varicella, or chickenpox, is a highly contagious infection characterized by an exanthematous vesicular rash and systemic symptoms. Primary varicella in adults is highly uncommon and could result in severe complications within this population. These complications include encephalitis, skin infections, pneumonia, and pneumonitis [7-9]. Of note, these complications are more common in adults. The primary causes of death associated with primary varicella infections in adults include pneumonia and encephalitis [10].

For this reason, an accurate diagnosis is critical to prevent life-threatening complications in this group of patients. Importantly, although primary varicella is uncommon in adults and even more so in the case of immunocompetent patients, family physicians should be well trained to recognize the characteristic cutaneous lesions of varicella and dictate adequate management for these patients to prevent severe complications. Therefore, this article presents an uncommon case of primary varicella in an immunocompetent adult patient during a recent varicella outbreak in Mexico City. We also deliver key messages on making an adequate differential diagnosis with other similar dermatoses such as herpes zoster (HZ). In this article, we also reinforce the importance of immunization in preventing re-emergent diseases and diminishing the complications attributed to these disorders in the population as a critical element within primary care management.

## Case presentation

We present the case of a 43-year-old woman who attended the dermatology clinic complaining of a recently appearing pruritic dermatosis. The patient reported that her symptoms started four days ago with headaches, malaise, myalgias, chills, and febricula, followed a day later by the appearance of a generalized rash predominantly found on her back. The patient denied any relevant medical history apart from being diagnosed with primary hypertension five years before her visit, which was managed with captopril. The patient did not consume any other medication. She had only one male sexual partner and was using an intrauterine device (IUD) for contraception.

**Figure 1:**
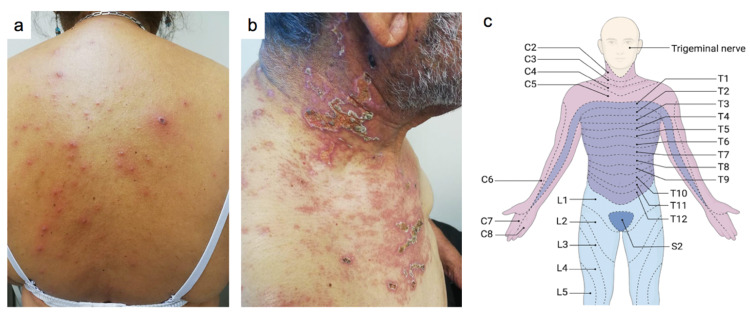
Representative images of the dermatoses of the patient and her husband (a) Rash composed of vesicles with erythematous bases, papules, and crusts within the dorsal region. (b) Localized rash characterized by the presence of small blisters and crusts. (c) Representation of the main dermatomes within the superior body segment. Image credit: The author, Eder Luna-Ceron, drew Figure 1c with the help of the BioRender platform.

On physical examination, the patient was febrile (38.5ºC) and presented a generalized rash composed of macules, vesicles with erythematous bases, and crusts (Figure 1, Panel a). The patient denied previous history of varicella infections and vaccination against varicella during her infancy. Her immunization record also confirmed this information. Further interrogation revealed that the patient's husband also presented a similar rash a week previously to her visit (Figure 1, Panel b). Notably, her husband's cutaneous examination revealed the presence of a painful rash characterized by small erythematous vesicles and crusts appearing predominantly in the region covered by the C2 to C5 dermatomes, and the rest of his examination remained normal (Figure 1, Panels b and c).

Further neurological examination, including Kernig's and Brudzinski's signs, was normal in the female patient. Additionally, the cardiovascular and respiratory exams did not show any abnormal findings. Laboratory evaluation is summarized in Table 1.

**Table 1:** Laboratory results of the patient on the day of the examination Results are expressed in values, and normal or abnormal results are noted in parenthesis. HIV: Human immunodeficiency virus; Ig: Immunoglobulin; AST: Aspartate aminotransferase; ALT: Alanine aminotransferase; hs-CRP: High-sensitivity C-reactive protein; ISR: immune status ratio.

Parameters	Results
HIV rapid testing	Negative
Varicella-zoster IgG antibody	31.2 ISR (Negative)
Varicella-zoster IgM antibody	5.2 ISR (Positive)
AST	22 U/L (Normal)
ALT	23 U/L (Normal)
hs-CRP	0.7 mg/L (Normal)
Procalcitonin	0.03 ng/mL (Normal)

Viral serology results demonstrated that the patient had an acute varicella infection. Inflammatory plasmatic markers and serum liver function tests were within normal limits. Further investigation for immunodeficiency, including human immunodeficiency virus (HIV) testing was completed without significant findings. As the patient did not show any signs of complications, the patient was given only symptomatic treatment, including second-generation antihistamines (loratadine) and acetaminophen for the following days. Notably, no antiviral therapy was added, given that the patient's rash was present for more than 72 hours. The patient received close follow-up for one week, showing complete remission of the symptoms. Regarding cutaneous lesions, only a few small fibrous scars were present in the back, while the rest of the lesions present in the first visit was absent. After three months of follow-up in the dermatology clinic, the patient showed complete resolution of the rash, no relapses, and an absence of complications related to her primary infection.

## Discussion

Based on the patient's age group, our primary diagnostic approach was to evaluate pruritic vesicular rashes commonly found in adults as potential differential diagnoses [9,11-13]. Epidemiology-wise, dermatoses consisting of pruritic vesicles in the adult population limit the possible pathologies, which are further described in Table 2.

**Table 2:** Main differential diagnoses representing dermatoses consisting of pruritic vesicles in the adult population HSV: Herpes simplex virus; VZV: Varicella-zoster virus.

Characteristics	Primary Varicella	Herpes Zoster	Dermatitis Herpetiformis	HSV	Dyshidrotic Eczema	Contact Dermatitis
Epidemiology	Mainly during childhood, most commonly in unvaccinated children	Mainly in older adults and patients with immunodeficiency	Adolescent and adults (15-40 years old), more frequent in males	Children and adults	Young adults	Children and adults
Physiopathology	Primary infection	Reactivation and multiplication of latent VZV that persisted within trigeminal and dorsal root ganglia following varicella	Genetic predisposition, autoimmune, associated with gluten sensitivity and celiac disease	Reactivation triggered by stress, immunodeficiency, and trauma	Unknown- multifactorial	Type IV hypersensitivity reaction (T-cell mediated)
Distribution	It begins on the face and scalp and progresses to spread to the trunk and the rest of the body	Unilateral following dermatomal distribution, disseminated in immunocompromised patients	Bilateral and symmetrical; can manifest in elbows, dorsal forearms, knees, buttocks, back, shoulders, and scalp without mucosal involvement	Oral/labial, vermilion border, genitals, and buttocks; disseminated lesions in patients with immunodeficiency	Hands and lateral aspect of fingers	Linear distribution localized where contact with allergen took place
Lesions	Scattered, they progress from rose-colored macules to papules, vesicles, pustules, and crust; rashes in all different stages	Erythematous maculopapular rash and vesicles within the affected dermatome	Grouped vesicles and papules, tense and with herpetiform appearance; followed by erosions, excoriations, and post-inflammatory hyperpigmentation, but most commonly heals without scarring	Painful vesicles that progress to a crust within a week	Symmetric eruption of vesicles may coalesce into bullae; usually persist for several weeks until desiccation and desquamation	Intensely pruritic dermatitis, characterized by erythema and vesicles in more severe cases
Symptomatology	Fever, malaise, anorexia, and pruritus	Pain in the distribution of the dermatome; pain is excruciating burning, tingling, itching, or stabbing	Intensely pruritic	Prodrome of pain, tingling, and burning 24 hours prior to rash development	Prodromal itching followed by abrupt onset of intensely pruritic vesicles	Pruritic and burning lesions

The patients’ clinical findings and interrogation revealed prodromal symptoms of pruritus, which can be associated with dyshidrotic eczema, dermatitis herpetiformis, herpes simplex virus (HSV), HZ, and primary varicella [9,11-13]. However, the rest of the prodromal symptomatology present in the patient such as malaise, headache, chills, myalgias, and fever centered our attention on infectious causes [9,12]. Furthermore, the topographical distribution of the lesions allowed us to rule out other non-infectious diagnoses. For example, dyshidrotic eczema can be ruled out by its distinctive distribution limited to the palms and interdigital spaces [13]. Although dermatitis herpetiformis can show a similar topography to the rash found in the patient by appearing in the torso, upper extremities, back, and buttocks, it can be ruled out by its tendency to be symmetrical and bilateral [11].

Another pruritic dermatosis that varies in distribution and may overlap with this clinical scenario is contact dermatitis as its location depends on where the allergen came in contact with the patient. Although not all cases are severe enough to cause vesicles, when it does, it tends to follow a linear pattern as opposed to the classic grouped appearance found in primary varicella, HZ, dermatitis herpetiformis, and HSV [14]. Given these features, contact dermatitis was ruled out as the possible diagnosis.

At this point, the possible diagnosis was centered on skin infections associated with the virus from the herpes family. In this sense, HSV tends to be localized to the vermilion border, genitals, or buttocks but may vary depending on the severity and immunocompetence of the patient, which, if compromised, may lead to a disseminated infection [12]. Additionally, the vesicular rash produced by HSV can progress to the formation of crusts as seen in the reported case [12]. However, we ruled out HSV as the diagnosis because its lesions are significantly painful in contrast with the non-tender lesions found in the patient [12].

Varicella and HZ (shingles) are distinct clinical entities caused by a single member of the herpesvirus family, varicella-zoster virus (VZV) [15]. Varicella usually develops during childhood, caused by the primary VZV infection [9]. In addition, it most commonly affects immunocompetent children (<five-years old) who are unvaccinated [9,16]. On the other hand, the rates of primary varicella in adults are rare, and it is usually a result of an immunodeficient state [17]. Clinically, its characteristic rash begins on the face and scalp and rapidly spreads to the trunk and the rest of the body. The lesions are scattered, but they progress from rose-colored macules to papules, vesicles, pustules, and crusts. The most critical clinical hallmark of primary varicella is the rash in all stages [17]. Systemic symptomatology such as fever and pruritus are mild to moderate, and long-term complications are rare in younger patients. Invasive group A streptococcal infections are a possible complication in immunocompetent patients [9]. However, varicella can be life-threatening in adults and immunocompromised patients of any age, such as pneumonia, hepatitis, encephalitis, hemorrhagic complications (mild febrile purpura to severe purpura fulminans), and myocarditis [17]. Given these complications, the cornerstone for prevention is vaccination.

As seen in this case, the presence of the characteristic lesions in different stages suggested the diagnosis of primary varicella. Additionally, the previous history of non-vaccination against varicella in this patient and the recent contact with a patient with HZ increased the clinical suspicion for this diagnosis. Furthermore, since immunodeficiency represents a significant risk factor for this infection, common immunodeficiency causes such as HIV infection or primary immunodeficiencies were also ruled out as direct causes. Furthermore, the differential diagnosis with HZ should also be considered, given the patient's age.

In contrast to primary varicella, HZ has a different clinical presentation. It is characterized by unilateral dermatomal pain and rash resulting from reactivation and multiplication of latent VZV that persisted within the trigeminal and dorsal root ganglia following a primary varicella infection [9,18]. The central lesions are an erythematous maculopapular rash and vesicles within the affected dermatome [19]. VZV reaches the skin via the sensory nerve from the single ganglion in which latent VZV reactivates and not by viremia [9]. It is most developed in older adults and immunocompromised patients [19]. The main clinical differences between HZ and primary varicella infection are further summarized in Table 3.

**Table 3:** Differences between herpes zoster and primary varicella VZV: Varicella-zoster virus.

Characteristics	Herpes Zoster	Primary Varicella
Epidemiology	Mainly older adults and immunocompromised patients	Mainly during childhood, most commonly in unvaccinated children
Physiopathology	Reactivation and multiplication of latent VZV that persisted within neurons following varicella	Primary infection
Distribution	Unilateral dermatomal pain and rash result from reactivation and multiplication of latent VZV that persisted within neurons following varicella	It begins on the face and scalp and progresses to spread to the trunk and the rest of the body.
Lesions	Erythematous maculopapular rash and vesicles within the affected dermatome	Scattered, they progress from rose-colored macules to papules, vesicles, pustules, and crusts. Rashes in all different stages.
Symptomatology	Pain in the distribution of the dermatome. Pain is excruciating burning, tingling, itching, or stabbing.	Fever, malaise, anorexia, and pruritus
Complications	Postherpetic neuralgia, bacterial superinfection, zoster gangrenous, pneumonitis, hepatitis, esophagitis, gastritis, pericarditis, cystitis, arthritis, meningoencephalitis, transverse myelitis, deafness, and cranial nerve palsies	Pneumonia, hepatitis, encephalitis, hemorrhagic complications (mild febrile purpura to severe purpura fulminans), invasive infections, arteritis, and myocarditis
Histopathology	Intranuclear inclusion bodies and multinucleated giant cell	Intranuclear inclusion bodies and multinucleated giant cell
Prophylaxis	Vaccination	Vaccination

Additionally, pain following the dermatome distribution is one of the main characteristics, and it is described as an excruciating burning, tingling, itching, or stabbing pain [20]. The pain can persist and become chronic neuropathic pain, known as postherpetic neuralgia [19]. Other common complications include bacterial superinfection, zoster gangrenous, pneumonitis, hepatitis, esophagitis, gastritis, pericarditis, cystitis, arthritis, meningoencephalitis, transverse myelitis, deafness, and cranial nerve palsies [9].

The clinical diagnosis for HZ was unlikely, given the patient’s presentation, who did not present pain or sensory abnormalities before the appearance or during the rash spreading. Also, the lesion topography, which did not follow a delimited dermatome area, made HZ an incompatible diagnosis. However, the clinical presentation of the patient’s husband is an excellent example of the HZ hallmarks previously mentioned. The serology results in this patient revealed positive immunoglobulin M (IgM) titers and negative immunoglobulin G (IgG) titers for VZV, thus confirming the presence of acute primary varicella infection and the absence of previous immunization.

Adults presenting with lesions compatible with primary varicella should undergo a complete neurological examination since diffuse encephalitis is one of the most severe complications in this group of patients [9]. Usually, these disorders can develop in the first week after the rash starts [21]. This disorder's clinical manifestations include delirium, focal neurological signs, meningeal inflammation signs, or seizures [22]. In this regard, the patient did not show signs of central nervous system (CNS) involvement in our case. However, since these clinical manifestations can start late after the rash, special counseling about these signs and symptoms was made and verified during the follow-up.

Similarly, adult patients presenting with primary varicella have a higher incidence of pneumonia. The early identification of this complication is critical since mortality rates as high as 30% have been reported with this clinical entity [23]. The main risk factors for pneumonia in primary varicella include a history of smoking, pregnancy or immunosuppression, and male sex [24]. In this regard, the patient from our case did not present any of the previously listed risk factors. This entity should be suspected in patients with progressive dyspnea, dry cough, or hemoptysis, and a chest radiograph should be ordered to confirm lung involvement. In the presence of these complications, the use of antiviral agents such as acyclovir has been the standard to increase the resolution rates. In our case, the patient did not show any respiratory symptoms, and respiratory physical examination remained normal even in the first follow-up appointment.

Hepatitis is another complication that should be identified promptly in adults with primary varicella [25]. It is essential to consider that immunosuppressed patients have a higher risk of this complication, usually presenting poor clinical outcomes [25]. However, mild transaminase elevation has been described even in healthy individuals with varicella [9]. Particular attention should be given to patients with two-fold or three-fold the upper normal rank value. These patients have a higher risk of hepatitis-associated complications such as disseminated intravascular coagulation (DIC) or gastrointestinal bleeding [25]. Of note, cases of fulminant acute hepatic failure, although rare, have been reported. In our case, the patient presented liver function tests within normal limits.

Immunocompetent adults with primary varicella can receive antiviral therapy with acyclovir given the evidence from a randomized clinical trial, demonstrating that this therapy can improve pruritus and significant reduction of the affected area. However, these significant effects were only seen in patients receiving therapy by the first day of rash appearance [26,27]. It is important to note that patients with systemic complications should receive antiviral therapy [26]. As seen in this case, the patient presented after more than 24 hours of rash spreading. Additionally, the patient did not present any systemic complications. Due to these factors, symptomatic therapy and close follow-up were considered for this patient.

## Conclusions

In conclusion, we reported the case of an adult patient with primary varicella, immunocompetent and unvaccinated, infected by a patient with HZ. After a full evaluation and follow-up, possible complications were ruled out. Any adult presenting with primary varicella infection requires an early diagnosis and prompt treatment to avoid any of the complications previously mentioned. A key point is a detailed contact history, physical examination, and the necessary lab work. Also, differential diagnoses with HZ infection should be considered due to their similarities and different repercussions. Given the seriousness of the complications, it is essential to emphasize the importance of prevention, which in this case can be obtained through vaccination. Primary care physicians should prioritize preventive measures such as vaccination to diminish the incidence of preventable diseases and their complications.
